# Cold Atmospheric Plasma-Activated Composite Hydrogel for an Enhanced and On-Demand Delivery of Antimicrobials

**DOI:** 10.1021/acsami.3c01208

**Published:** 2023-04-11

**Authors:** Nishtha Gaur, Bethany L. Patenall, Bhagirath Ghimire, Naing T. Thet, Jordan E. Gardiner, Krystal E. Le Doare, Gordon Ramage, Bryn Short, Rachel A. Heylen, Craig Williams, Robert D. Short, Toby A. Jenkins

**Affiliations:** † Department of Chemistry, Lancaster University, Lancaster LA1 4YB, U.K.; ‡ Department of Chemistry, University of Bath, Bath BA2 7AY, U.K.; § Glasgow Dental School, School of Medicine, University of Glasgow, Glasgow G12 8TA, U.K.; ∥ Microbiology Department, Lancaster Royal Infirmary, University of Lancaster, Lancaster LA1 4YW, U.K.; ⊥ Department of Chemistry, The University of Sheffield, Sheffield S3 7HF, U.K.

**Keywords:** cold atmospheric plasma, plasma jet, composite hydrogels, drug delivery, reactive oxygen and nitrogen species, antimicrobial, biofilms, gentamicin

## Abstract

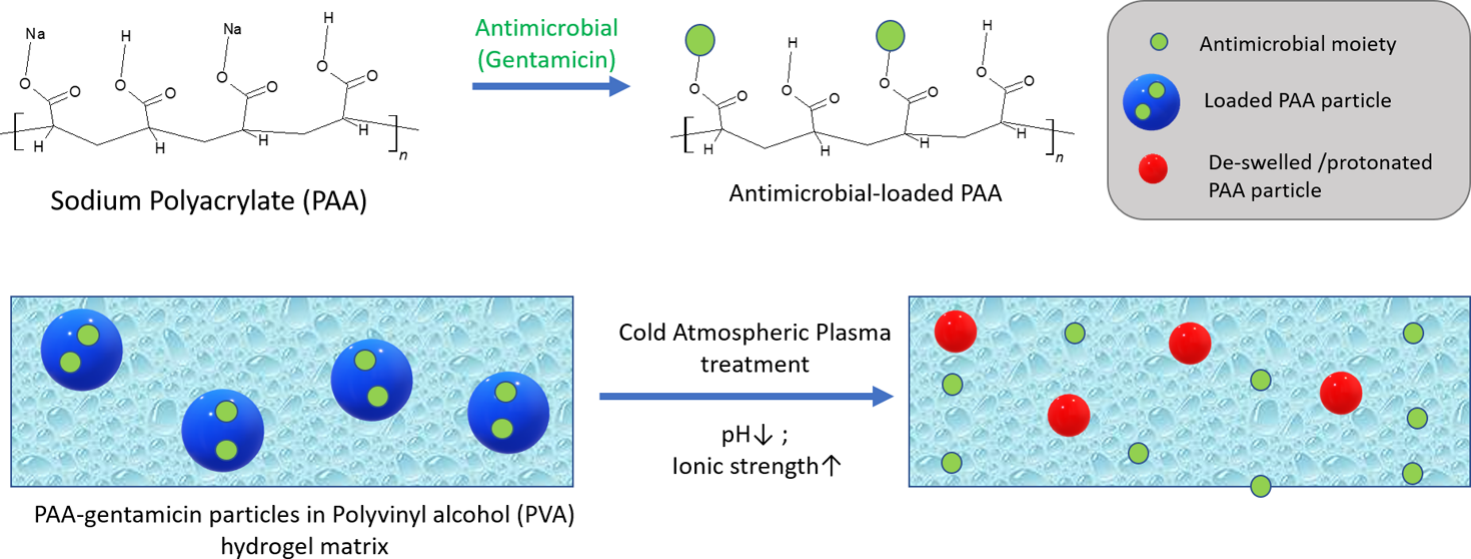

We present the concept of a versatile drug-loaded composite hydrogel that can be activated using an argon-based cold atmospheric plasma (CAP) jet to deliver both a drug and CAP-generated molecules, concomitantly, in a tissue target. To demonstrate this concept, we utilized the antibiotic gentamicin that is encapsulated in sodium polyacrylate (PAA) particles, which are dispersed within a poly(vinyl alcohol) (PVA) hydrogel matrix. The final product is a gentamicin-PAA-PVA composite hydrogel suitable for an on-demand triggered release using CAP. We show that by activating using CAP, we can effectively release gentamicin from the hydrogel and also eradicate the bacteria effectively, both in the planktonic state and within a biofilm. Besides gentamicin, we also successfully demonstrate the applicability of the CAP-activated composite hydrogel loaded with other antimicrobial agents such as cetrimide and silver. This concept of a composite hydrogel is potentially adaptable to a range of therapeutics (such as antimicrobials, anticancer agents, and nanoparticles) and activatable using any dielectric barrier discharge CAP device.

## Introduction

Hydrogels are frequently used in wound dressings, as they provide an occlusive matrix and keep the wound hydrated, which aids wound healing. Moreover, hydrogels have proven utility in drug delivery, for example, nicotine and fentanyl patches.^1^ One challenge in designing hydrogels for drug delivery to skin, wounds, or other topical areas is to trigger the drug release following a specific stimulus.^2^ It is often undesirable to have a slow passive release from the hydrogel dressing if the drug being eluted is present in concentrations below its therapeutic concentration, e.g., in the case of an antimicrobial below its minimal inhibitory concentration (MIC). MIC is defined as the lowest concentration of a drug capable of inhibiting the growth of the microbe(s).

We describe a composite hydrogel system that is designed to work in response to cold atmospheric plasma (CAP) to provide a antimicrobial dose above the MIC. This approach extends to the utility of CAP, which is a relatively an exciting new treatment modality for wound disinfection and cancer eradication.^3^

Composite hydrogels are materials wherein a mixture of chemically and physically different hydrogel polymers is contained within a single material matrix to utilize the favorable chemical properties (but poor structural properties) of one polymer with another structurally superior polymer.^4^ In this study, the functional polymer is a super-absorbent polymer, sodium polyacrylate (PAA), that is combined with a secondary polymer, cryo-crosslinked poly(vinyl alcohol) (PVA). PAA-based polymers, both homopolymer dispersions and copolymers, have attracted interest as potential drug delivery vehicles (in addition to their principal application in hygiene products), due to their high swellability and sensitivity to pH, wherein exposure to a solution below the p*K*_a_ of the carboxylate (ca. pH 4.2) causes a rapid gel collapse and drug release.^5^ In this study, PAA particles are used to “store” the antibiotic gentamicin and release it upon triggering by CAP, while the PVA provides the structural matrix in which the PAA particles are embedded.

There is a growing body of literature on the direct application of CAP in the control of microbial biofilms within wounds.^6−9^ Briefly, CAP is an ionized gas generated at room temperature and is composed of a “cocktail” of reactive oxygen and nitrogen species (RONS) such as hydrogen peroxide (H_2_O_2_), hydroxyl radicals (^•^OH), nitric oxide (NO), nitrite (NO_2_^–^), nitrate (NO_3_^–^), and peroxynitrite (ONOO^–^), which (dependent on the context) have all been considered biologically beneficial. It is worth highlighting that besides RONS, other components of CAP such as electric fields and charged species may also contribute (alone or in combination with these RONS) to complex biological effects.^10−12^

The composition of CAP and concentrations of CAP-generated RONS can be controlled by modifying the CAP operating parameters such as gas flow rate, electrodes, gas type, treatment distance from the target, and exposure time.^13^ Despite generally thought to be safe, it cannot be the case that all the RONS produced by CAP are beneficial in all circumstances, for example, some concerns have been raised about the use of CAP regarding genotoxicity due to the presence of the highly reactive ^•^OH.^14^ As previously shown by Gaur et al., introducing a hydrogel film between the CAP source and target, during CAP treatment, can reduce the genotoxic effects of CAP while still allowing the delivery of beneficial RONS such as H_2_O_2_.^14^

**Figure 1:**
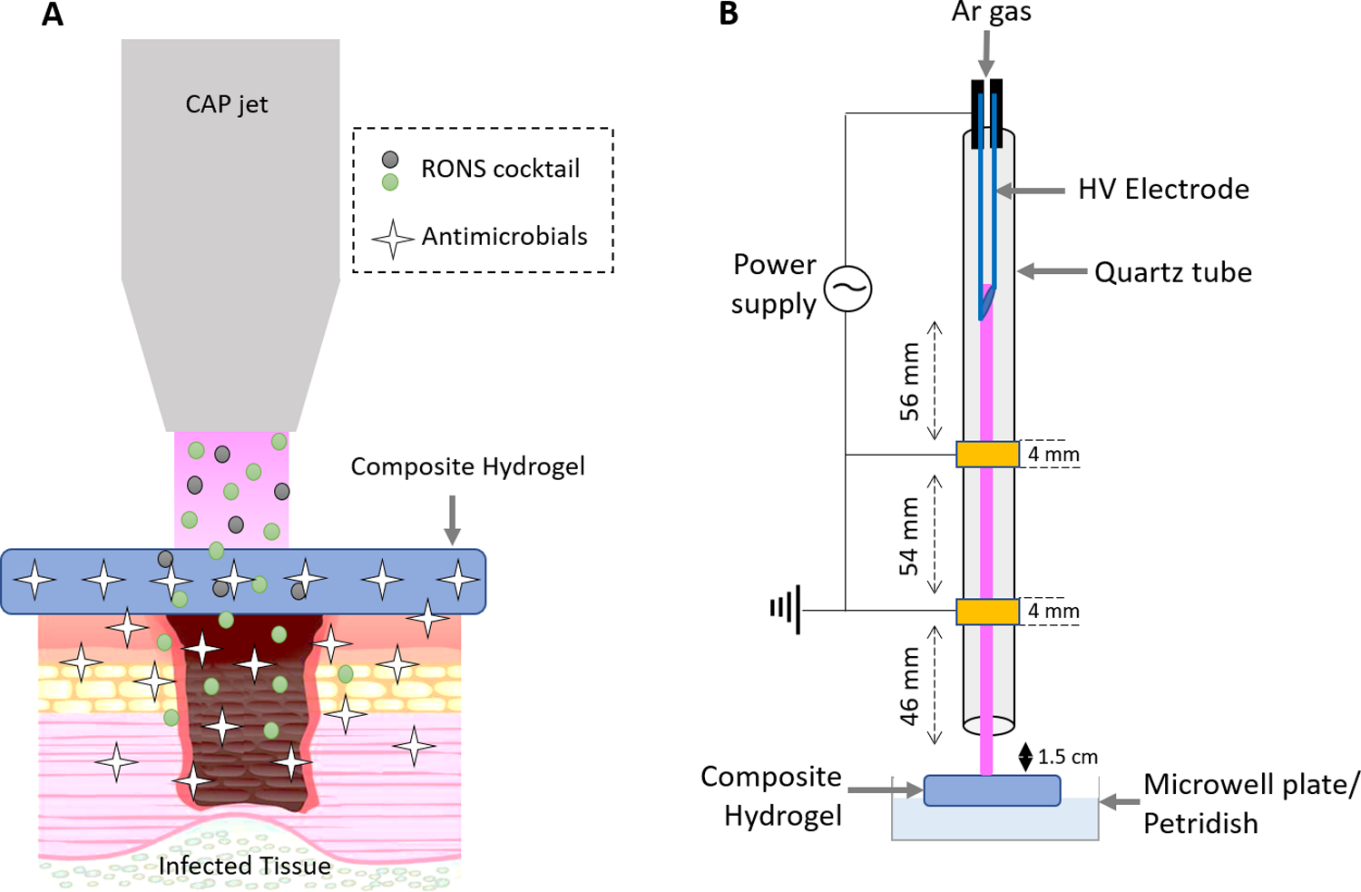
(A) Illustration of the concept of CAP-activated composite hydrogel therapy for an infected tissue. (B) Schematic of the Ar CAP jet operated at a gas flow rate of 1 SLPM, peak-to-peak voltage of 7 kV, and frequency of 23.5 kHz. The composite hydrogel is placed on a target (aqueous solution in a microwell plate or microbes in a Petri dish). The schematic is for illustrative purposes only and does not represent the exact laboratory treatment conditions.

The rationale for this study was to create a versatile, easy-to-prepare, and potentially clinically deployable hydrogel matrix, which can be activated using a CAP jet to release its therapeutic (herein, an antimicrobial) cargo in a controllable and reproducible way, while using low-cost polymers and not requiring complex, synthetic chemical steps for synthesis. The gentamicin-loaded PAA is triggered by a combination of pH and osmolarity to “collapse” and pump out the cargo (see below). As shown in Figure 1A, the concept is to place the antimicrobial-loaded composite hydrogel on top of the infected tissue, followed by exposure to a CAP jet, thus allowing an effective delivery of the therapeutic and desirable beneficial RONS deep into the tissue. As the first proof of principle, the release of the cationic antibiotic gentamicin was demonstrated, with release (where possible) being quantified using a colorimetric assay. Microbiological assays were used to demonstrate the efficacy against the bacterial species *Staphylococcus aureus* and *Pseudomonas aeruginosa*, which are commonly isolated from infected chronic wounds.^15^

The composite hydrogels were prepared by dissolving gentamicin sulfate (Sigma-Aldrich) in deionized (DI) water at a concentration of 1 mg mL^–1^. Once fully dissolved, PAA particles (SAVIVA, BASF), at a concentration of 1% w/v, were added to the gentamicin sulfate solution and left at room temperature for 30 min. The PAA polymer absorbs the gentamicin solution and swells, thus resulting in gentamicin-loaded PAA particles. These particles were then washed with copious amounts of water and ethanol using a Büchner funnel under vacuum to wash away any unbound gentamicin. The washed gentamicin-PAA particles were dried by repeated freeze–thaw cycles under low pressure using liquid nitrogen and washing with ethanol. Finally, the gentamicin-loaded PAA particles were dried at 60 °C under vacuum until a dry powder was achieved. 100 mg of the dried gentamicin-loaded PAA particles was mixed with 5 g of PVA (Sigma-Aldrich) and ground together in a pestle and mortar to obtain a homogeneous powder. The gentamicin-PAA-PVA powder is dissolved in 100 mL of DI water and maintained in a water bath at 95 °C for 1 h. 20 mL of the gentamicin-PAA-PVA solution was then added to a 9 cm-diameter Petri dish and spread evenly. The solution of gentamicin-loaded PAA in the PVA gel (referred to as gentamicin-PAA-PVA hydrogels thereafter) was stored at −20 °C until frozen and then defrosted at 25 °C. This freeze–thaw process was repeated twice more to enable cryo-crosslinking of PVA. Discs of 10 mm were prepared using a biopsy punch and placed on top of the target (buffer solution/bacterial lawn/biofilms), followed by CAP treatment. The CAP jet used for this study to activate the composite hydrogel is illustrated in Figure 1B. It is equipped with a stainless-steel electrode, which served as the high-voltage (HV) electrode and has an inner diameter (ID) of 0.6 mm and outer diameter (OD) of 0.9 mm. The HV electrode is enclosed inside a quartz tube (ID = 1.5 mm; OD = 3.0 mm) with two copper ground electrodes (width = 4 mm) wound on the quartz tube at positions of 56 and 110 mm below the tip of the HV electrode. The length of the quartz tube below the end of the second ground electrode was 46 mm. This arrangement maximizes H_2_O_2_ production, while keeping the effluent temperature low to process thermally sensitive materials. The treatment distance between the tip of the quartz tube and the target was 1.5 cm, and the CAP jet was stationary unless otherwise stated. The jet was operated by purging argon (Ar) gas at a flow rate of 1 standard liter per minute (SLPM) through the quartz tube and applying 7 kV at 23.5 kHz to the HV electrode using a sinusoidal power supply (PVM-500, amazing1.com). Voltage and current waveforms were measured at the HV electrode using a high-voltage probe (Pintek Electronics Co. Ltd.) and a current probe (Pearson Electronics Inc.), respectively, with the waveforms recorded on an oscilloscope (Siglent Technologies Co. Ltd). Optical emission spectra (OES) were recorded using a HR4000CG-UV-NIR spectrometer (Ocean Optics) equipped with an optical fiber of 600 μm diameter. The fiber was placed 4 mm away from the outside of the quartz tube for the CAP jet.

**Figure 2:**
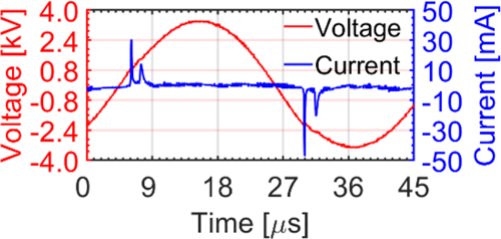
Electrical characterization of the CAP jet including the current–voltage waveforms.

**Figure 3:**
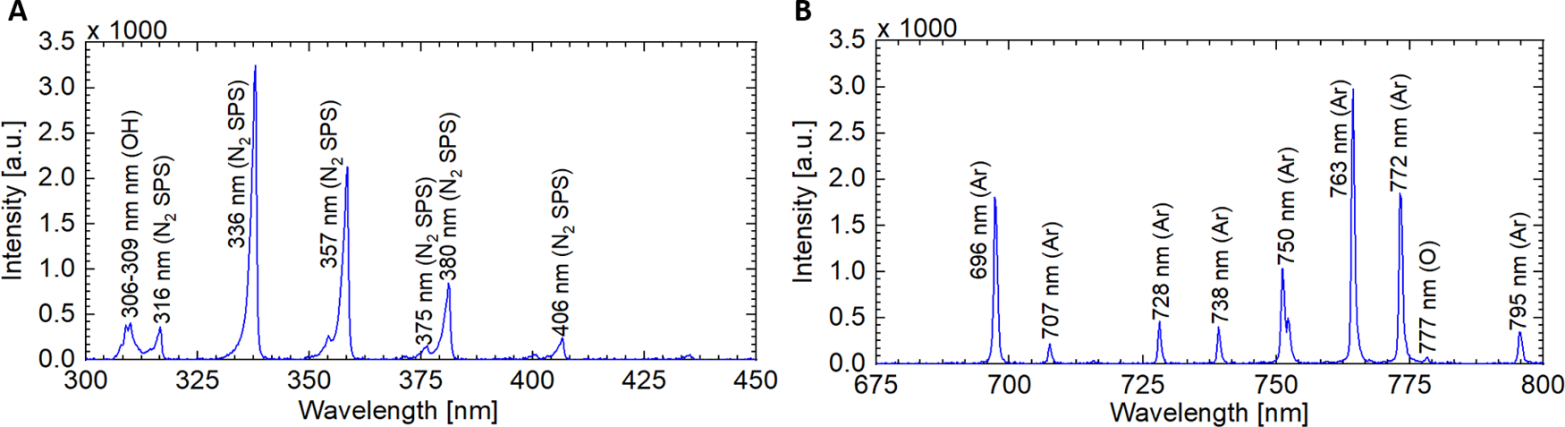
OES recorded for the CAP jet in the range of (A) 300–450 nm and (B) 675–800 nm depicting the emissions from ^•^OH, N_2_ SPS, Ar*, and O.

The detailed analysis of physical (electrical and optical) and chemical (RONS production) characteristics of the CAP jet can be found in our previous publication.^16^ Briefly, a sinusoidal voltage waveform with two current peaks formed between the HV electrode and each of the two ground electrodes (Figure 2). The OES in the range of 300–450 and 675–800 nm is shown in Figure 3A,B, respectively. As shown in Figure 3A, the typical OES included emission lines for ^•^OH (306–309 nm) and several bands of the nitrogen second positive system (N_2_ SPS) at 316, 337, 357, 375, 380, and 406 nm. In the range of 675–800 nm (Figure 3B), the emission is mainly composed of excited Ar (Ar*) from 696 to 796 nm. Emission from atomic oxygen (O) is also observed at 777 nm.^17,18^

**Figure 4:**
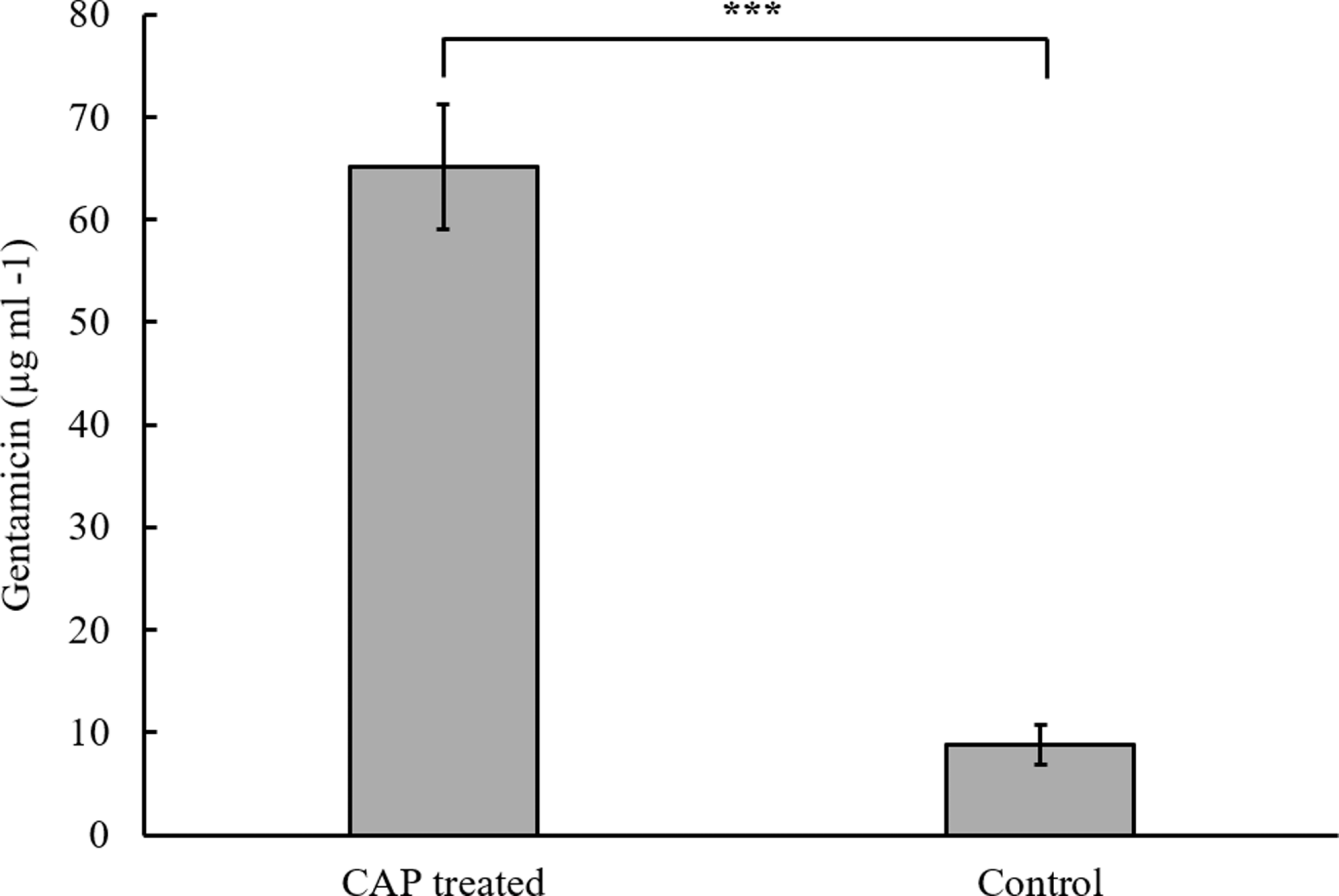
Gentamicin release from the composite hydrogel following a 2 min CAP jet treatment. Error bars represent standard deviation (*n* = 3). Student’s *t*-test was carried out to assess statistical significance, *p* < 0.001.

The first step was to investigate the delivery of gentamicin from the composite hydrogel following CAP treatment. Ninhydrin solution (Sigma-Aldrich) was used to quantify the release of gentamicin, as per the manufacturer’s instructions (S6; Supporting Information), from the composite hydrogel both before (passive release) and after (triggered release) the CAP treatment.^19^ Gentamicin is an aminoglycoside antibiotic, which has a net positive charge at pH 7, due to the protonation of its two secondary amine groups (p*K*_a_ = 8.8 and 9.9).^20^ Ninhydrin reacts with primary and secondary amines to form a purple color precipitate, which is detected spectrophotometrically at 540 nm.^21^ To measure the gentamicin release, gentamicin-PAA-PVA hydrogels were dipped in 1 mL of 1× phosphate buffer solution (PBS) in a well of a 48-microwell plate (Sigma-Aldrich) and treated with the CAP jet for 2 min, followed by incubation for 24 h at ambient conditions. A calibration curve (Figure S1; Supporting Information) was constructed with known concentrations of gentamicin in PBS using ninhydrin assay and used to calculate the concentration of gentamicin in PBS from the CAP-treated gentamicin-PAA-PVA hydrogels. Figure 4 shows the passive and triggered release of gentamicin from the gel. After 2 min of treatment with the CAP jet, approximately 65 μg mL^–1^ gentamicin was released from the gel into the PBS, which is significantly higher than the passively released 1.1 μg mL^–1^. The MIC of gentamicin against most clinically important bacteria (within skin wounds) is in the range of 1–4 μg mL^–1^ for most clinical strains of *S. aureus* and *P. aeruginosa.*(22)

**Figure 5:**
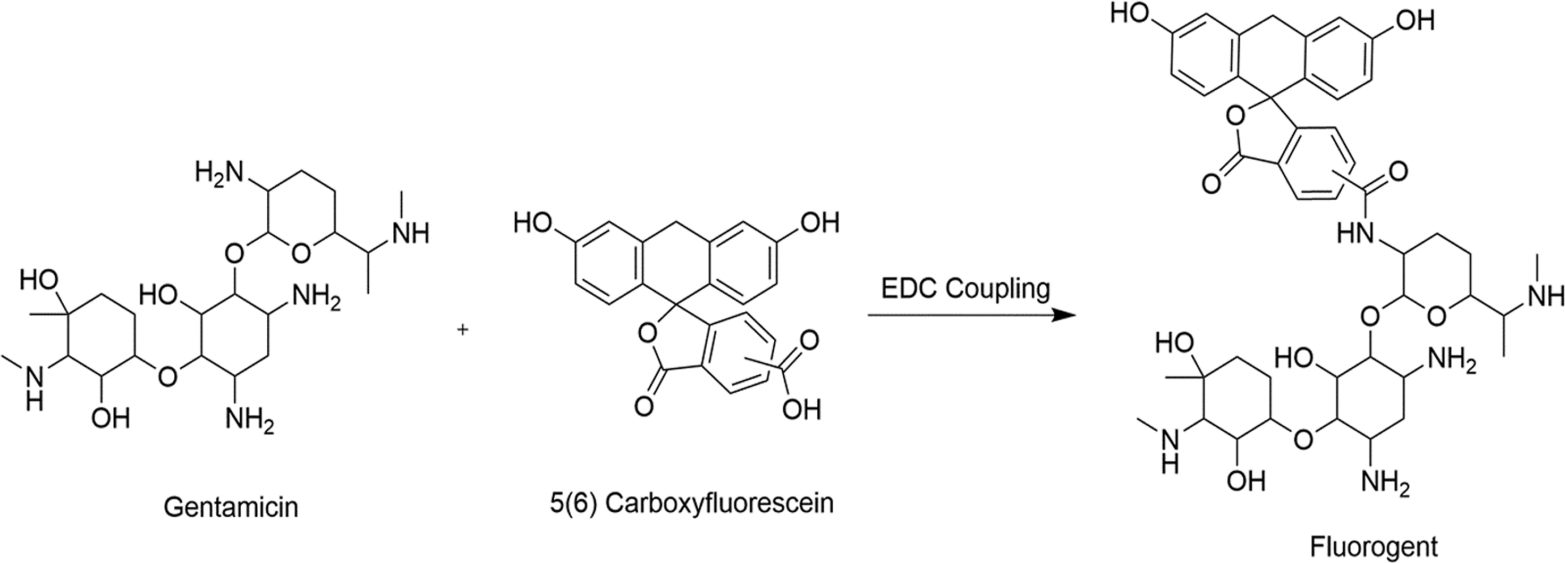
EDC/NHS coupling of carboxyfluorescein to free amines on gentamicin. The most likely product based on steric crowding and amine p*K*_a_ is shown but is likely a mixture of products.

**Figure 6:**
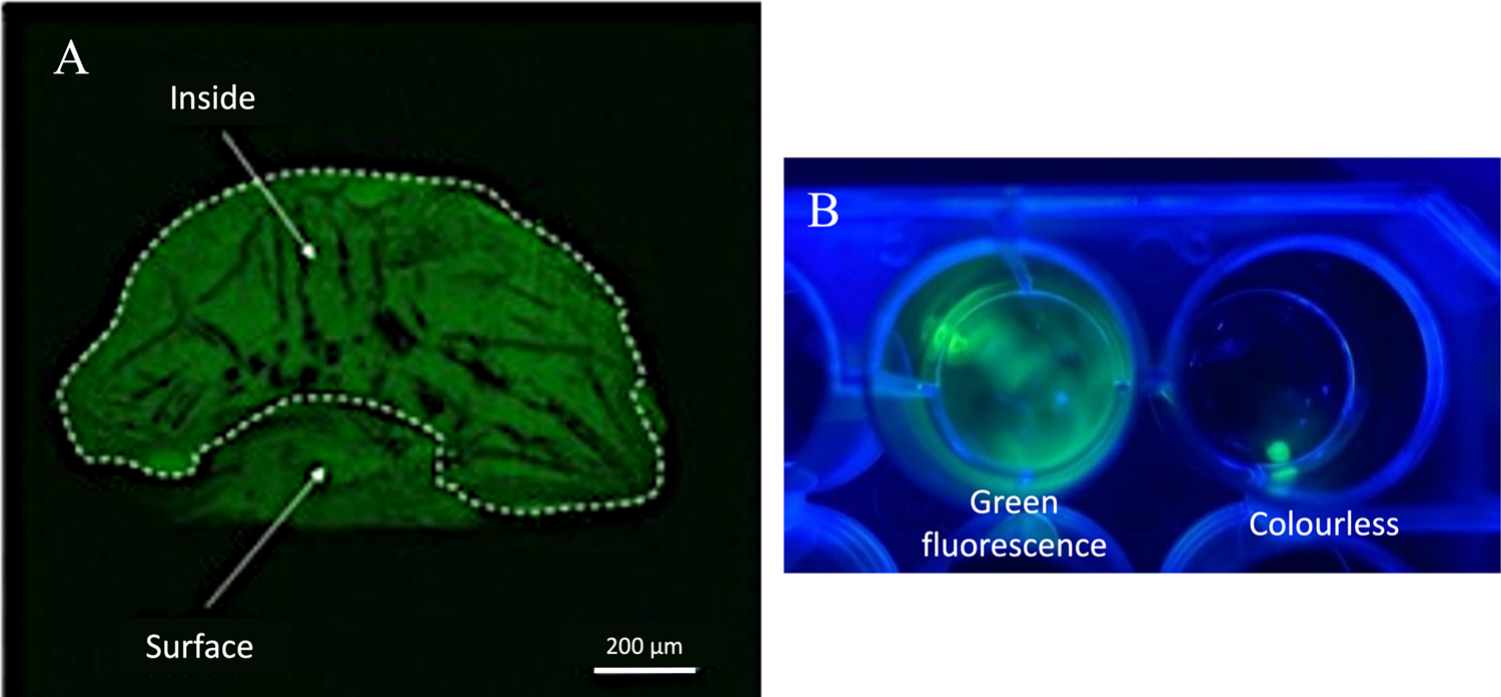
(A) Fluorescent microscope image of fluorogent-loaded PAA, cut in half. (B) Release of carboxyfluorescein from the particle (left) and retention of the fluorogent in the particle (right) when immersed in water.

To better understand the distribution of gentamicin within the PAA particle, gentamicin was fluorescently tagged with carboxyfluorescein using 1-ethyl-3-(-3-dimethylaminopropyl)carbodiimide/N-hydroxysuccinimide (EDC/NHS) coupling (Figure 5), using 1:1 molar ratio of the two reactants, herein referred to as “fluorogent.” The “fluorogent” was allowed to diffuse into PAA particles, followed by washing of the particles with water (following the same procedure used for gentamicin-loaded particles). The particle was then sectioned using a razor blade and imaged under a fluorescent microscope (LSM800) at an excitation and emission wavelength of 490 and 520 nm emission, respectively. As gentamicin has a number of amines susceptible to coupling to the fluorophore, it is likely the fluorogent product is a mixture of a number of products. Figure 6A shows that the fluorogent was distributed homogeneously inside the PAA particle and not localized on or near the particle surface. Immersion of the fluorogent-loaded PAA particle in water shows retention of the dye in the particle compared with out-diffusion of carboxyfluorescein-loaded PAA (Figure 6B), which being an anionic dye has no cationic groups to coulombically interact with PAA particles and was used here as a negative control. To test the versatility of PAA-PVA particles with other therapeutic agents, we repeated this experiment with silver (Ag^+^), where Ag+ is a potent antimicrobial. The successful loading data of Ag^+^ in PAA-PVA are shown in the Supporting Information (Figure S3).

**Figure 7:**
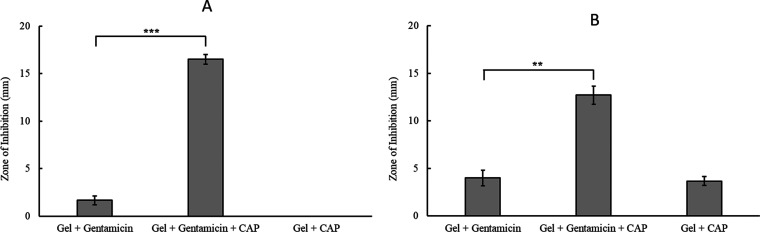
ZOI of gentamicin-PAA-PVA hydrogels with and without CAP activation and PAA-PVA gels (no gentamicin) with CAP activation for two bacterial species: (A) *P. aeruginosa* and (B) *S. aureus*. Error bars represent standard deviation (*n* = 3), and a one-way ANOVA was carried out to calculate statistical significance, *p* < 0.001.

The Kirby–Bauer (KB) test was used to assess the susceptibility of bacteria to CAP-triggered release of gentamicin. In the KB test, a sterile disc soaked in the test compound is placed on a bacterial lawn and a zone of bacterial growth inhibition (ZOI) is created by the out-diffusion of the agent under the test. In this case, a modified form of the KB test was used (detailed in Section S3; Supporting Information) with bacterial lawns of *P. aeruginosa* (PAO1) and *S. aureus* (H560). As shown in Figure 7, different types of composite hydrogel discs were tested—untreated gentamicin-PAA-PVA hydrogels (Gel + gentamicin), CAP-activated gentamicin-PAA-PVA hydrogels (Gel + gentamicin + CAP), and CAP-activated PAA-PVA hydrogels without gentamicin (Gel + CAP).

As shown in Figure 7A for *P. aeruginosa*, the ZOI measured for the untreated gentamicin-containing gel is 1.67 mm, which is thought to have resulted from the passive release of gentamicin from the hydrogel. However, CAP-activated gentamicin gels resulted in a 16-fold increase in the ZOI (16.5 mm), showing the effectiveness of CAP-mediated gentamicin release, with a possible synergy between the CAP-produced RONS and gentamicin. Interestingly, the unloaded gels (i.e., without gentamicin) had no effect on the *P. aeruginosa* bacterial lawn (ZOI = 0 mm) even after CAP activation, thus suggesting that the concentration of antimicrobial species (RONS) generated by the CAP was well below the RONS MIC for *P. aeruginosa* and/or an antimicrobial is essential for its eradication. This trend of enhanced killing by the CAP activation of gentamicin-loaded gels was also observed for *S. aureus*, with a ZOI of 12.7 mm formed in the case of CAP-treated gentamicin gels and approximately 4 mm in the case of both untreated gentamicin gels and CAP-treated gels without gentamicin (Figure 7B).

The formation of the ZOI in gentamicin gels (Figure 7), even without CAP, suggests that some passive release of gentamicin takes place, albeit at low levels. It is assumed that a drug (gentamicin) is retained within the PAA particles via coulombic interactions. It is likely that the more the cationic groups on the drug, which interact with the carboxylate groups in the PAA, the better the retention of the drug (lower passive release). This hypothesis was tested by encapsulating a large antimicrobial peptide, polymyxin-B, with 15 cationic amines in its structure vs. 5 cationic amines in gentamicin. The KB test results (Figure S6; Supporting Information) show a significantly lower passive release of polymyxin-B, suggesting that a large additive effect from multiple cation–anion interactions helps retain the drug in the PAA.

**Figure 8:**
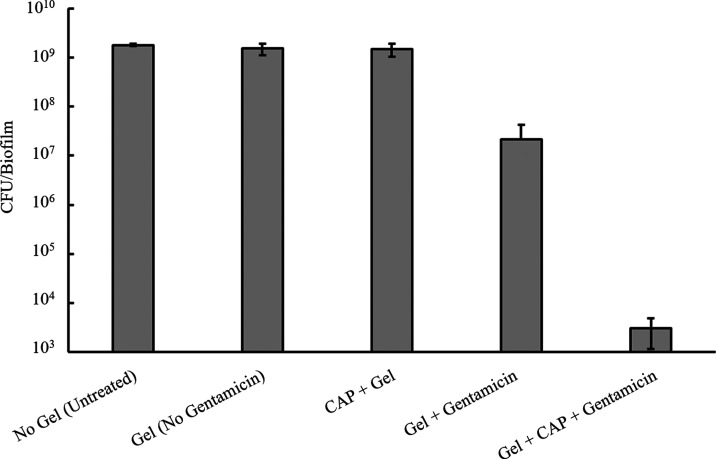
Viable cell count of 8 h *P. aeruginosa* (PAO1) biofilms after 18 h of incubation with composite hydrogels loaded with water (with and without CAP activation) and gentamicin-loaded composite hydrogels (with and without CAP activation) relative to untreated control biofilms. The error bars show standard deviation (*n* = 3).

To test the gel system against a more clinically relevant model, early-stage (8 hr) *P. aeruginosa* biofilms were prepared on a nanoporous polycarbonate membrane (Whatman) atop of culture media.^23^ (See Section S5 of the Supporting Information for the detailed protocol.) Composite hydrogels (with or without gentamicin) were then placed on top of the biofilms and treated for 5 min with CAP. The untreated biofilm, i.e., no gel and no CAP exposure, was used as a negative control. Following treatment, the biofilm was stripped and the viable cell count was enumerated (CFU/biofilm). As shown in Figure 8, compared to the untreated, a 2-log reduction in viable cells was observed when biofilms were exposed to gentamicin-PAA-PVA gels, even without the application of the CAP jet. This is likely due to the passive release of gentamicin. However, a significantly higher (>5-log) reduction in viable cells was observed when the biofilm is exposed to a CAP-activated gentamicin-PAA-PVA composite gel, thus highlighting the synergistic action of RONS and gentamicin in biofilm eradication. To ensure that the results observed were a product of CAP-stimulated gentamicin release and not simply from the CAP-delivered RONS, a composite gel without gentamicin was applied to the biofilms with and without CAP activation. No reduction in CFU was observed in either case.

The exact mechanism of CAP-triggered release of the antimicrobial gentamicin from the PAA-PVA composite gel is still under investigation. However, a plausible mechanism involves the role of pH and ionic strength (IS). The coulombic interaction of the carboxylate anions in PAA and positively charged groups of gentamicin is believed to be the principal mechanism by which the antimicrobial is bound to the composite gel matrix. The deswelling of PAA particles is pH- and ionic strength-driven. A 10-fold increase in conductivity from 50 to 1080 μS cm^–1^ and a decrease in pH by ca. 1 pH unit from pH 5.08 to 3.9 were observed after CAP treatment of DI water. (Detailed study on conductance and pH can be found in Figure S4 of the Supporting Information.) As shown in Figure 3, CAP is a rich source of ^•^OH, which can combine to form H_2_O_2_ (eq 1).^24^
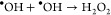
1Furthermore, the OES also reveals the presence of excited N_2_ species, which react with oxygen (O_2_) to form NO (eq 2).^25^ Upon solvation, NO produces NO_2_^–^ (eq 3), which can further react with H_2_O_2_ to form nitrates, NO_3_^–^ (eq 4).^24^ In an acidic environment, NO and H_2_O_2_ undergo a complex redox chemistry to form ONOO^–^, further contributing to lowering the pH.^25^
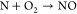
2
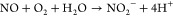
3
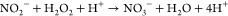
4This highlights that the gentamicin release from the hydrogel is not solely dependent on H_2_O_2_, which is considered the “major” species in CAP treatment, and that the presence of N_2_ species is significant.

This CAP-mediated change in IS and pH protonates the carboxylate groups on the PAA particle, causing a disruption in the coulombic attraction between PAA and gentamicin, at the same time reducing the interchain repulsion of the PAA chain, and thus leading to a collapse of the PAA particle and “pumping out” gentamicin in the surroundings. The released gentamicin is then delivered from the gel into the target by the action of CAP. It has been demonstrated previously by Szili et al. that CAP has the potential to drive the molecules (RONS) several millimeters deep into a gelatin-based tissue model.^26^ This is partly enhanced by CAP-initiated ultraviolet photolysis and electric field induced by the accumulated charges onto the target material.^27^ However, it is also plausible that the flux of CAP-generated H_2_O_2_ helps carry the released antimicrobial out of the gel and into the underlying water, but experiments to prove this are ongoing.

The versatility of the composite hydrogel system has been further tested by incorporating a cationic (nonantibiotic) antimicrobial, cetrimide, an antimicrobial peptide, polymyxin-B, and dendrimer nanostructures. CAP-activated cetrimide gels exhibited effective antimicrobial activity when tested in a KB assay (Figure S5; Supporting Information). For polymyxin-B, as discussed previously, an excellent retention of the drug was observed, with minimal evidence of passive release (Figure S6; Supporting Information). The limitation of (the composite hydrogel) requiring a cationic drug can be overcome by employing a positively charged drug-loaded carrier vehicle. We have demonstrated the utility of our system by demonstrating the loading and release of amine-terminated dendrimers (Figures S7 and S8; Supporting Information), vehicles for carrying drug molecules that may be uncharged or indeed negatively charged. The use of antimicrobial peptides and dendrimers in the composite hydrogels is subject to a current study and will be reported in more detail in the future.

**Figure 9:**
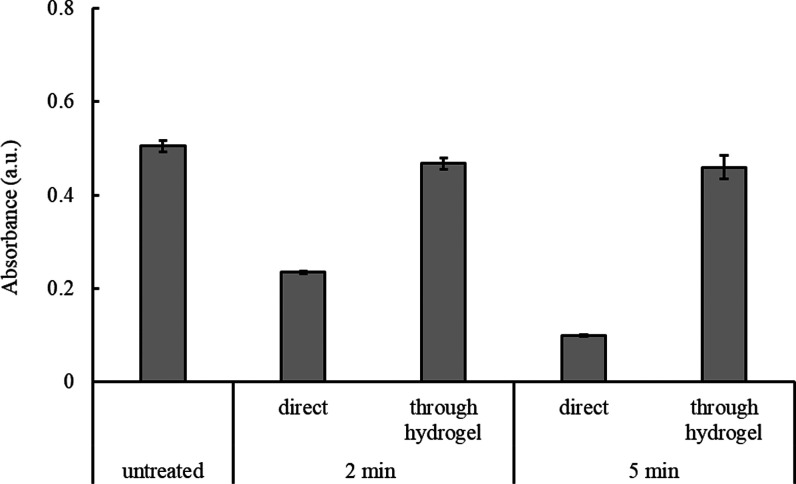
Absorbance values of MB solution at 664 nm measured before (untreated) and after CAP treatment for 2 and 5 min. CAP treatments of MB were conducted without (direct) and through the PVA hydrogel. Error bars show standard deviation (*n* = 3).

As stated previously,^14^ a key benefit of using a hydrogel during CAP treatments is its ability to extinguish the highly reactive genotoxic CAP components such as ^•^OH. To test this, ^•^OH production was qualitatively measured using methylene blue (MB) assay (Section S13; Supporting Information). Upon interaction with ^•^OH, MB undergoes oxidative degradation and decolorization, resulting in a decrease in absorbance values.^28^ As shown in Figure 9, upon direct treatment of MB, i.e., without intervention of the PVA hydrogel film, the absorbance of MB reduced from 0.5 (a.u.) to 0.2 and 0.09 after 2 and 5 min of CAP exposure, respectively. In contrast, for treatments through the hydrogel, the absorbance values were in a similar range to that of untreated MB even after prolonged CAP exposure of 5 min. The insignificant change in absorbance values after the introduction of the hydrogel proves the potential of the composite hydrogel to prevent the delivery of ^•^OH into the target. Overall, this result highlights the potential of the composite hydrogel to provide a safe means to deliver the drug moiety to the tissue target.

## Conclusions

In this article, we have demonstrated that a cationic antibiotic, gentamicin, can be encapsulated within PAA particles and that when the loaded particles were dispersed in a secondary PVA gel matrix, gentamicin can be released “on demand” by exposing to a CAP jet. The release is most likely triggered via the protonation of carboxylate groups in the PAA and a RONS flux. We show an enhanced antimicrobial effect against planktonic and early-stage biofilms, potentially due to an improved gentamicin delivery as well as delivery of RONS, which are themselves antimicrobial.^29^ Previous (as well as current) work has shown the importance of using a hydrogel to remove potentially genotoxic components of the CAP jet^14^ and raise the oxygen tension in the tissue.^26,30−32^ Raising the oxygen tension in this way should both inhibit the growth of anaerobic bacteria and also promote healing.^19^ The treatment of deep chronic wounds requires targeted antimicrobial therapy with small-molecule antimicrobials needing to be delivered deep into the wound matrix. On-going work demonstrates that such encapsulation/release can be achieved with a broad range of antimicrobial moieties including silver ions, cetrimide (a quaternary ammonium cation), and the antimicrobial peptide polymyxin-B as well as dendrimers. The concept of CAP-treated hydrogels for drug delivery in cancer treatment was recently discussed by Živanić et al.; however, the hydrogel they proposed is CAP-treated in a non-crosslinked state before turning into a 3D network upon injection.^33^ We believe that the approach shown here is novel, can be adapted to any type of CAP device, and has the potential to be utilized with a broad range of therapeutics, opening up opportunities in other indications such as cancers and skin-related autoimmune conditions.

## Supporting Information Available

Details of biological materials used in the study; bacterial culture and biofilm formation; ninhydrin assay; KB assay; scanning electron microscopy, energy dispersive X-ray analysis; and composite hydrogel loaded with cetrimide, polymyxin-B, and dendrimers (PDF)

## Abbreviations

MIC: minimal inhibitory concentration
CAP: cold atmospheric plasma
PAA: sodium polyacrylate
PVA: poly(vinyl alcohol)
RONS: reactive oxygen and nitrogen species
H_2_O_2_: hydrogen peroxide
^•^OH: hydroxyl radicals
NO: nitric oxide
NO_2_^−^: nitrite
NO_3_^−^: nitrate
ONOO^−^: peroxynitrite
DI: deionized
HV: high voltage
ID: inner diameter
OD: outer diameter
Ar: argon
SLPM: standard liters per minute
OES: optical emission spectra
N_2_ SPS: nitrogen second positive system
Ar*: excited Ar
O: atomic oxygen
PBS: phosphate buffer solution
EDC: 1-ethyl-3-(-3-dimethylaminopropyl)carbodiimide
NHS: N-hydroxysuccinimide
Ag^+^: ionic silver
KB: Kirby–Bauer
ZOI: zone of bacterial growth inhibition
IS: ionic Strength
O_2_: oxygen
