# Supplementary material for: Cold Atmospheric Plasma-Activated Composite Hydrogel for an Enhanced and On-Demand Delivery of Antimicrobials
Source: ACS Appl Mater Interfaces. 2023 Apr 11;15(16):19989–96. doi: 10.1021/acsami.3c01208 (PMC10141252; doi:10.1021/acsami.3c01208)
Supplement: Supplementary file 1 — am3c01208_si_001.pdf [file am3c01208_si_001.pdf]

SUPPORTING INFORMATION

A Cold Atmospheric Plasma-activated Composite Hydrogel for an enhanced and on-demand delivery of Antimicrobials

*Nishtha Gaur[† *‡], Bethany L. Patenall[§ β‡], Bhagirath Ghimire[† ≈], Naing T. Thet[§], Jordan E. Gardiner[§], Krystal E. Le Doare[§], Gordon Ramage[+], Bryn Short[+], Rachel A. Heylen[§], Craig Williams[^], Robert D. Short[† ~], Toby A. Jenkins[§]*

[†]Department of Chemistry, Lancaster University, Lancaster, LA1 4YB, UK.

[§]Department of Chemistry, University of Bath, Bath, BA2 7AY, UK.

[+]Glasgow Dental School, School of Medicine, University of Glasgow, Glasgow, G12 8TA, UK

[^]Microbiology Department, Lancaster Royal Infirmary, University of Lancaster, Lancaster, LA1 4YW, UK

[~]Department of Chemistry, The University of Sheffield, Sheffield, S3 7HF, UK

S1: Media, buffers and stock solutions used in this study:

	**Abbreviation**	**Supplier**
Muller Hinton Agar	MHA	Sigma
Muller Hinton broth	MHB	Sigma
Tryptic soy agar	TSA	Sigma
Luria-Bertani agar	LBA	ThermoFischer
Glycerol	-	Sigma
Phosphate Buffer Saline	PBS	Sigma

S2: Protocol for bacterial strains and overnight cultures

Pseudomonas aeruginosa (*P. aeruginosa*) strain PAO1 and *Staphylococcus aureus* (*S. aureus*) strain H560 were acquired from the Jenkins group collection at the University of Bath. Strains were maintained on 15 % (v/v) glycerol at -80°C and revived when required. *S. aureus* was cultured on TSA and *P. aeruginosa* on LBA to obtain single colonies. Plates were incubated at 37°C for 24 h.

S3: Kirby-Bauer (KB) test assay

Single colonies were inoculated into 10 mL of MHB and incubated for 18 h at 37°C with agitation at 200 rpm. Cultures were spun down at 10,000 rpm and pellet resuspended in PBS, subcultures were made in 10 mL of MHB and grown to OD_{600} 0.5. 100 μL of bacterial culture was added to MHA and spread to create to create a lawn. Gel discs of 10 mm in diameter were placed in the centre of the lawn using sterile forceps. Gels were then activated or no activated as specified. After treatments plates were incubated statically for 24 h at 37°C. The zone of inhibition (ZOI) was measured using a ruler and blank corrected to the diameter of the individual disc to account for any variation.

S4: Minimum Inhibitory Concentration (MIC)

Single colonies were inoculated into 10 mL of MHB and incubated for 18 h at 37°C with agitation at 200 rpm. Cultures were spun down at 10,000 rpm and pellet resuspended in PBS, subcultures were made in 10 mL of MHB and grown to OD_{600} 0.01. 200 μL of gentamicin antibiotic solution was added into the wells and serially diluted into 100 μL, two-fold across a 96-well plate into MHB. 100 μL of bacteria was then added to each well of these wells. As a negative control and blank, 200 μL of bacteria solution and broth was added to separate wells, respectively. Plates were incubated for 18 h at 37°C and MIC was read by eye and defined by the lowest concentration that inhibits bacterial growth denoted by lack of bacterial turbidity.

S5: Bacterial biofilm formation

Sterile discs (diameter 19 mm) were placed onto BHIA and UV sterilized for 10 minutes. 20 μL of artificial wound fluid (AWF) (1:1 peptone water to fetal calf serum) was aliquoted onto the discs and spread and left to dry. Bacterial overnights were spun and resuspended in PBS and corrected to 0.01 OD_{600}, 30 μL of bacterial subculture was then added onto the disc. These were then incubated at 37°C for 8 h and then removed. Discs were cut to a diameter of 20 mm to completely cover the biofilm and treated accordingly. Biofilms were then incubated for a further one hour to allow for killing effect of released antimicrobial. Biofilms were then removed from the BHIA using sterile forceps and placed into 5 mL of PBS in a 15 mL falcon tube. Tubes were then vortexed for 1 minute and sonicated for 15 minutes. This is repeated once. This is then serially diluted in PBS (0 – 10^{-7}) and plated out on TSA or LB plate to enumerate viable cells.

S6: Ninhydrin assay for gentamicin release

Gentamicin sulphate (Sigma) was prepared to a range of concentrations 100-1000 μg/mL in pH7.4 buffer solution. 500 μL of antibiotic into an Eppendorf to which add 500 μL of 1% ninhydrin solution was added. These are then incubated in 95°C heat block for 45 minutes.

After cooling it down to room temperature, 200 μL was dispensed into wells of 96-well plate and absorbance was measured at 540 nm.

S7: Gentamicin calibration curve

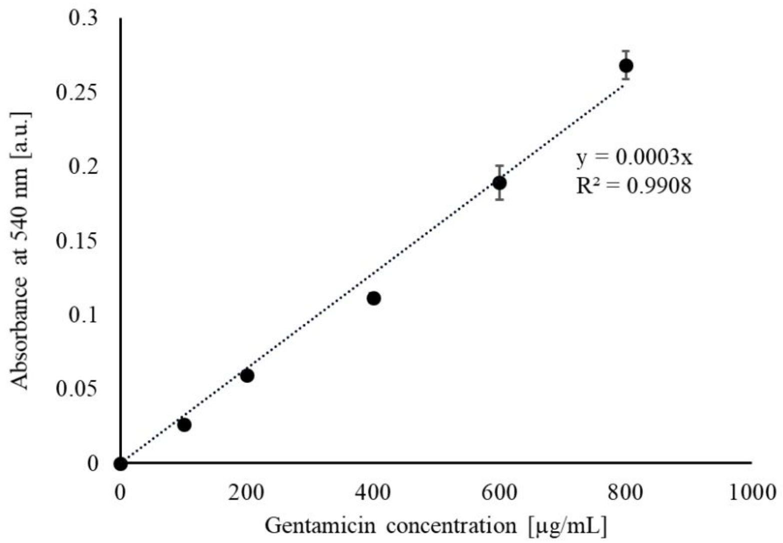

Figure S1. Standard curve for the quantification of gentamicin using ninhydrin. Error bars represent the standard deviation (N=3).

S8: Loading of silver (Ag^+) in composite hydrogel system

Ag^+ loading in PAA particles were characterized by using scanning electron microscopy-energy dispersive X-ray (SEM-EDX). It was demonstrated by loading 1mg ml^{-1} silver nitrate in 10 mg of PAA (the protocol was same as the gentamicin loading described in the manuscript). The silver nitrate loaded dry particles were cut into half and analyzed using JEOL

JSM-7900F field emission scanning electron microscope (Figure S2). An EDX spectra was generated at a specific location (box-marked in Figure S2) as shown in Figure S3. A peak at 3 keV corresponding to Ag confirmed the presence of Ag in the PAA particle.

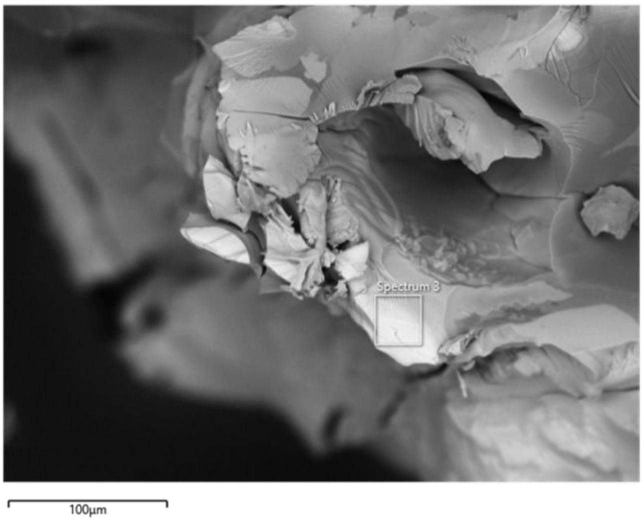

Figure S2. SEM image of the cross-sectioned PAA particle showing the specific area (marked as spectrum 3) on which the EDX analysis was performed.

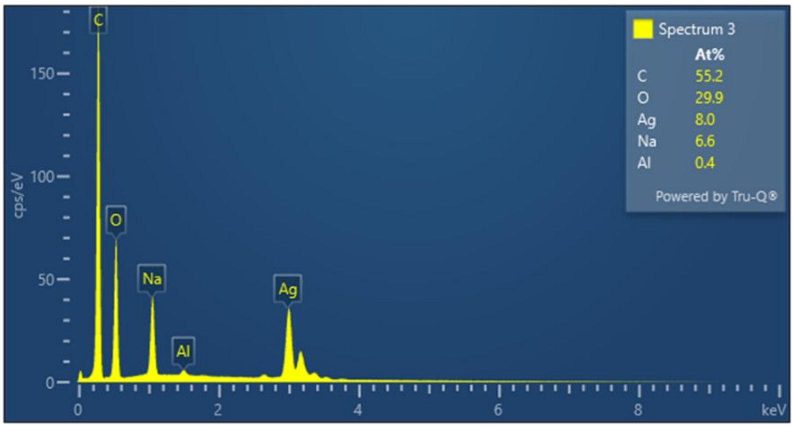

Figure S3. EDX spectrum from the cross-sectional surface of a silver nitrate-loaded PAA

particle. The spectra was obtained at an accelerating voltage of 10 kV, magnification of 320 times and a working distance of 10.1 mm.

S9: pH and Conductance change post CAP treatment

As shown in Figure S4A, a significant increase in conductance from ca. 15 to 119 µS cm^{-1} was observed as CAP treatment time increased. No change in conductance was observed n untreated water (control). On the other hand, pH value decreased from 7 to ca. 4 in a short treatment time of 4 minutes and further to ca. 3 after 20 mins of treatment (Figure S4B).

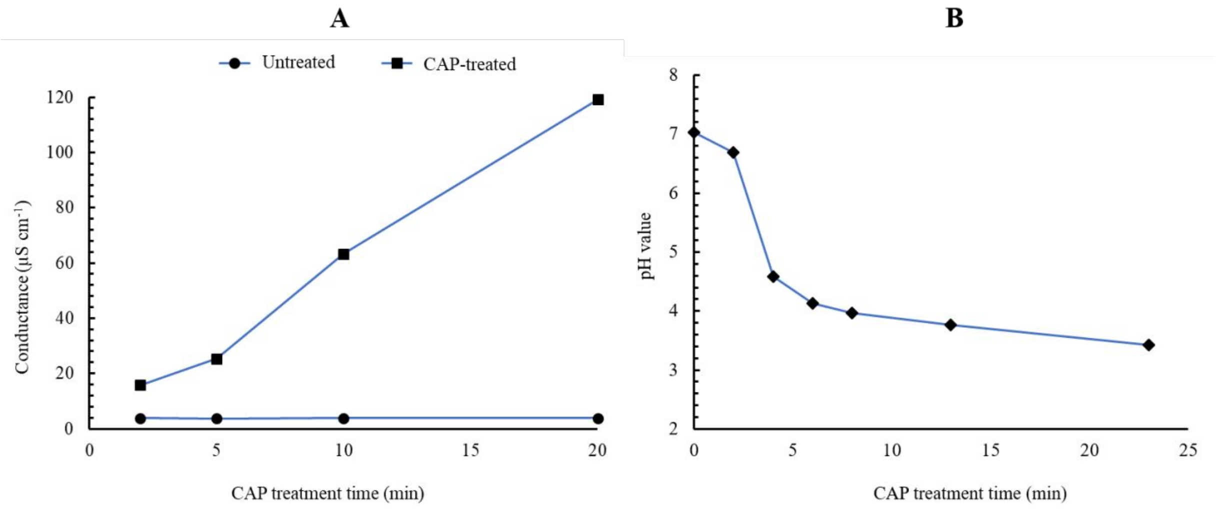

Figure S4. Change in (A) conductance and (B) pH values after CAP treatment of DI water

S10: ZOI of CAP-activated polymyxin-B and cetrimide-loaded PAA-PVA particles

Cetrimide or polymyxin-B solution at 10% or 0.1% w/v, respectively, was prepared in DI water before adding to 1% w/v PAA in a round bottom flask. This followed the same drug loading procedure described in the manuscript. KB test assay was performed as described in section

S3. ZOI of drug-loaded composite hydrogels (polymyxin-B or cetrimide gels) with and without CAP-activation was compared to unloaded composite gels (PVA/PAA Gel) with CAP-activation. As shown in Figure S5A, no *P. aeruginosa* killing was observed with PVA/PAA gel even after CAP treatment. However, addition of cetrimide or to the gels in case of both untreated and CAP-treated cetrimide gels leads to bacterial killing. However, with CAP action a significantly higher ZOI (15 mm) was observed, cf. cetrimide only gel (ZOI = 5mm). This highlights the role of CAP in driving the cetrimide through the gel. A similar effect was observed in *S. aureus* wherein cetrimide-loaded gels when activated with CAP exhibited maximum ZOI (Figure S5B). Polymyxin-B gels (Figure S6) result in a very low passive release, potentially because of stronger (additive) interaction with the PAA, as it has multiple cationic groups but larger ZOIs following CAP application.

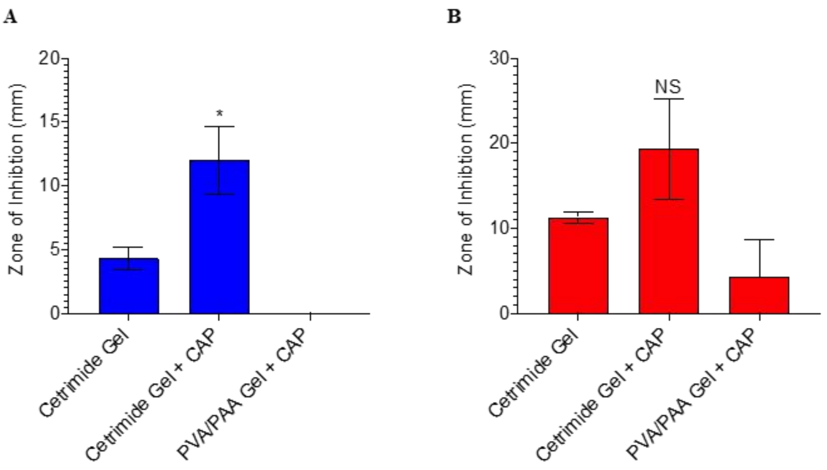

Figure S5. ZOI of cetrimide-loaded composite hydrogel (cetrimide gel) against (A) *P. aeruginosa* and (B) *S. aureus*. CAP treatment of both cetrimide gels and unloaded gels (PVA/PAA Gel) was compared. Error bars represent standard deviation (N=3) and a One-way ANOVA was carried out using GraphPad 8.0. (* = p<0.1).

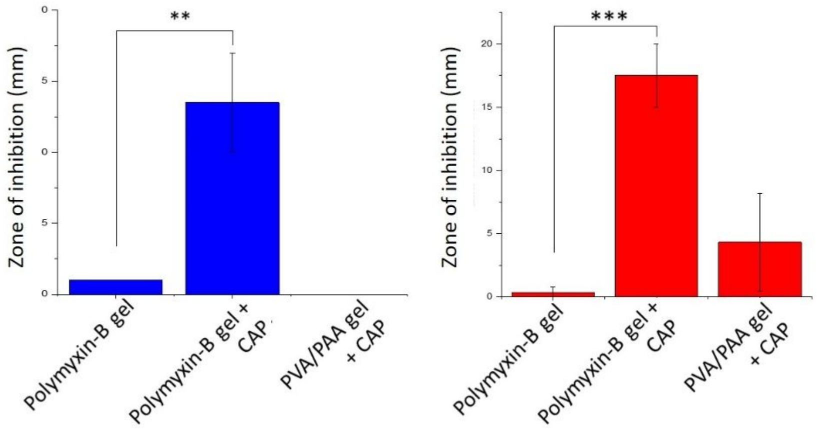

Figure S6. ZOI of polymyxin-B-loaded composite hydrogel (polymyxin-B gel) against (A) *P. aeruginosa* and (B) *S. aureus*. CAP treatment of both polymyxin-B gels and unloaded gels (PVA/PAA Gel) was compared. Error bars represent standard deviation (N=3) and a One-way ANOVA was carried out using GraphPad 8.0. (** means p<0.01; *** means p<0.001).

S11: Release of dendrimers from CAP-activated PAA-PVA particles

Generation 1 PAMAM dendrimer with ethylenediamine core (Sigma Aldrich) at a concentration of 0.213 mM in DI water was added to PAA particles (1% w/v) and left at room temperature for 1 hr. The particles were then treated with CAP jet (direct) and plasma-activated water (indirect). Blank and 0.1 mg/ml PAMAM solution was used as a negative and positive control, respectively. Ninhydrin assay was performed as described earlier. As shown in Figure S7, direct CAP treatment resulted in purple color formation indicating the release of dendrimers into the solution and interaction with ninhydrin reagent to form a purple color. Absorbance measurements indicate a high absorbance with direct CAP treatment c.f. untreated and indirect CAP treatment.

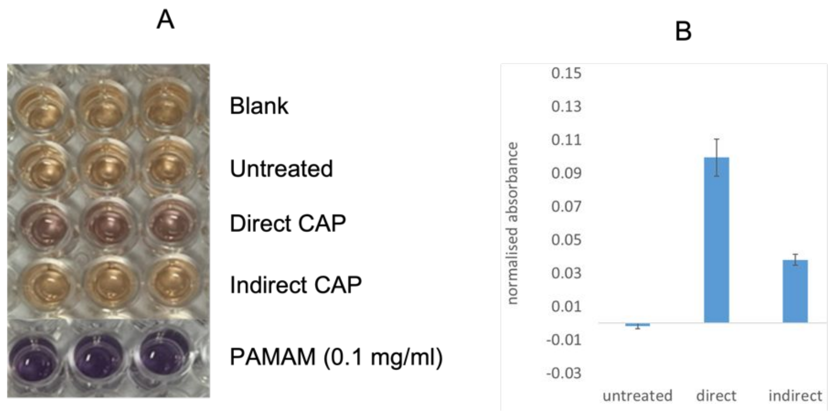

Figure S7. Ninhydrin assay performed on PAMAM-loaded PAA particles post CAP treatment. (A) A purple color formed after CAP-treatment; (B) Absorbance measurements of the

ninhydrin reagent.

S12: Encapsulation of anionic dye in PAMAM-loaded PAA particles

To validate whether PAMAM-loaded PAA can be used as a delivery vehicle for anionic agents (otherwise difficult to load in PAA particles), an anionic dye tartrazine (Sigma-Aldrich) was investigated. PAMAM:dye concentration was 1:10 in 0.1 g of PAA particles. The mixture was left at room temperature for 1 hr. The particles were then washed with copious amount of water over Buchner's funnel under vacuum. As shown in Figure S8, dye loaded in PAA particles (left) washed out completely as the particles fail to encapsulate the anionic agent. However, when trapped in the dendrimers (right), the dye remained trapped in the PAA particles.

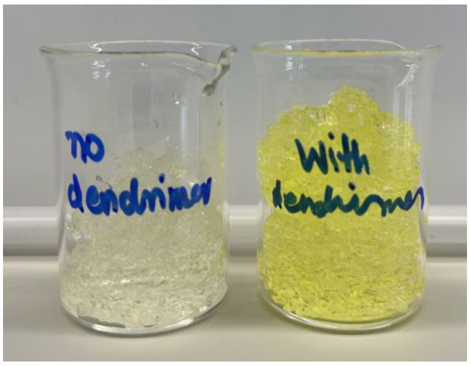

Figure S8. Proof of principle demonstrating the ability of PAA particles to encapsulate anionic dye - tartrazine in presence of dendrimers.

S13: Methylene blue (MB) assay for hydroxyl radical detection

Aliquot of 1 ml or 1.65 ml of MB solution (0.01 mg/ml in DI water) in a 48-well microwell plate was exposed directly or indirectly (through PVA hydrogel) to CAP jet, respectively. Immediately after the treatment, 100 μL of treated MB solution was dispensed into wells of 96-well plate and absorbance was measured at 664 nm.
